# Right Ventricular Subclinical Dysfunction in SLE Patients Correlates with Metabolomic Fingerprint and Organ Damage

**DOI:** 10.3390/metabo13070781

**Published:** 2023-06-22

**Authors:** Martino Deidda, Antonio Noto, Davide Firinu, Cristina Piras, William Cordeddu, Claudia Depau, Giulia Costanzo, Stefano Del Giacco, Luigi Atzori, Giuseppe Mercuro, Christian Cadeddu Dessalvi

**Affiliations:** 1 Department of Medical Sciences and Public Health, University of Cagliari, 09042 Monserrato, Italycadedduc@unica.it (C.C.D.); 2 Department of Biomedical Sciences, University of Cagliari, 09042 Monserrato, Italylatzori@unica.it (L.A.)

**Keywords:** metabolomics, 3D echocardiography, global longitudinal strain, systemic lupus erythematosus

## Abstract

Systemic lupus erythematosus (SLE) is a chronic inflammatory disease, and several studies have suggested possible early RV involvement. Aim of the study was to evaluate the 3D echo parameters of the right ventricle (RV) and the metabolomic profile to correlate both with SLE severity. Forty SLE patients, free of cardiovascular disease, were enrolled and the following 3D parameters were evaluated: the RV ejection fraction (RV-EF), longitudinal strain of the interventricular septum (Septal LS), longitudinal strain of the free wall (Free-LS) and the fractional area change (FAC). In addition, a metabolomic analysis was performed. Direct correlations were observed between TAPSE values and the RV 3D parameters. Then, when splitting the population according to the SDI value, it was found that patients with higher cumulative damage (≥3) had significantly lower FAC, RV-EF, Septal LS, and Free-LS values; the latter three parameters showed a significant correlation with the metabolic profile of the patients. Furthermore, the division based on SDI values identified different metabolic profiles related to the degree of RV dysfunction. The RV dysfunction induced by the chronic inflammatory state present in SLE can be identified early by 3D echocardiography. Its severity seems to be related to systemic organ damage and the results associated with a specific metabolic fingerprint constituted by 2,4-dihydroxybutyric acid, 3,4-dihydroxybutyric acid, citric acid, glucose, glutamine, glycine, linoleic acid, oleic acid, phosphate, urea, and valine.

## 1. Introduction

Systemic lupus erythematosus (SLE) is a systemic chronic disease characterized by the aberrant activity of the immune system that leads to a highly variable course and prognosis. SLE affects nearly 1.5 million people in the United States, and its prevalence is three- to four-folds higher in Black than in White females [1].

Cardiovascular diseases (CVD) represent the leading cause of morbidity and mortality in SLE. A particular challenge is pulmonary arterial hypertension (PAH) in SLE patients as a complication of their autoimmune disease, which has impacts on the quality of life and prognosis.

This serious complication has a prevalence ranging from 9.3% to 14% and is characterized by a progressive increase in pulmonary vascular resistance with vascular remodeling, right ventricular failure, intolerance to exercise, and death [2].

In this setting, right ventricular failure is the leading cause of death; the right ventricle’s (RV) ability to adapt to the increase in pulmonary vascular resistance associated with pulmonary arterial hypertension plays a crucial role in determining the survival and functional capacity of these patients [3].

A previous study showed an early and subclinical reduction in RV longitudinal function in SLE patients depending on their SLE organ damage score [4]. On the other hand, SLE pathophysiology, related to the cytokine asset, subtends the magnitude of organ damage [5].

Organ damage also involves the heart; increasing findings indicate myocardial inflammation as a pathophysiologic process leading to heart failure [6].

The complex inflammatory response related to cytokine effects contributes, together with the increased RV after-load due to autoimmune diseases such as SLE, to the RV (mal)adaptive remodeling that occurs in RV dysfunction, which is similar to what is known regarding left ventricle hypertrophy and failure [6].

Moreover, our group has already demonstrated the metabolomic utility in investigating cardiovascular diseases [7] and cardiac involvement in autoimmune pathologies [8].

On these bases, we performed a metabolomic analysis based on both ^1^H-Nuclear magnetic resonance spectroscopy (^1^H-NMR) and gas chromatography/mass spectrometry (GC/MS) to better clarify the metabolic milieu through which RV dysfunction develops. In addition, we used 3D speckle tracking echocardiography (STE) to evaluate the RV’s performance and correlated its systolic parameters with the metabolic profiles identified using metabolomic analyses.

## 2. Materials and Methods

We consecutively enrolled 40 SLE patients coming from the Clinical Immunology Outpatient Clinic of the AOU of Cagliari; among these, we selected subjects free of cardiovascular pathology—including coronary artery disease, hypertensive heart disease, cardiomyopathies, valvopathy greater than a moderate degree, previous deep vein thrombosis, or pulmonary embolism. Moreover, we enrolled in analysis those in whom the 3D acquisitions were of sufficient quality to guarantee adequate ventricular volume tracking during analysis.

The Institutional Ethics Committee (Azienda Ospedaliero—University of Cagliari) approved the study (Protocol PG/2015/1859, 2 February 2015), which was performed according to the Declaration of Helsinki. Participants, informed of the study aims and methodology, expressed written consent to enrollment before inclusion. All patients underwent clinical evaluation and 12-lead electrocardiogram; moreover, we performed echocardiography with the evaluation of standard systo-diastolic parameters and 3D acquisitions for the assessment of the following RV parameters: end-diastolic volume (VTD), end-systolic volume (VTS), ejection fraction (RV-FE), longitudinal strain of the SIV (Septal LS), longitudinal strain of the free wall (Free-LS), and the fractional area change (FAC). In addition, venous blood samples were collected from all patients for cytokine assays and metabolomic analysis.

### 2.1. Standard Echocardiography

We performed standard mono- and two-dimensional, as well as color Doppler echocardiography by measuring the ventricular volumes and thicknesses and calculating the left ventricle ejection fraction (LVEF) using the Simpson’s biplane method; a value ≤ of 50% was considered abnormal. Subsequently, to evaluate the diastolic function, we recorded the flow velocities of the pulsed Doppler in the four apical chambers view; moreover, we used tissue Doppler imaging (TDI) with the sample volume in the basal portion of the interventricular septum (IVS) and lateral wall. We evaluated the LV longitudinal function by measuring the following parameters of the mitral valve annulus: peak systolic velocity (S wave), peak velocity in proto-diastole (E′ wave), peak velocity of atrial contraction (A′ wave), and LV isovolumetric relaxation time (IVRT). Furthermore, we acquired the raw data using the STE technique.

### 2.2. The 3D Echocardiography

We acquired the “full-volume” 3D datasets using a complete matrix array transducer (Vivid E80, GE Healthcare, Boston, MA, USA) with the probe positioned in apical view; then, during a brief breath interruption, we carried out the acquisition. We used six electrocardiographic-gated consecutive heartbeats acquired during an end-expiratory apnea (multi-beat acquisition) to generate the whole volume. Subsequently, we verified the acquisition quality in 12-slice display mode to exclude stitching artefacts and ensure that the entire RV cavity and walls were included in the total volume with optimal RV border visualization. Data sets deemed adequate were digitally stored in raw data format and exported to a dedicated workstation equipped with commercially available software (4D RV-Function, TomTec Imaging Systems, Gmbh, Unterschleissheim, Germany) for offline analysis. Every RV full-volume 3D dataset automatically produced three standard views (planes): four-chamber, coronal, and sagittal. An operator traced the endocardial border at the end-diastole and end-systole for the three predefined RV planes, and then an automated border detection algorithm completed the tracking. When needed, the same operator corrected the endocardial borders frame-by-frame to minimize artefacts. Papillary muscles of the tricuspid valve, moderator band, and endocardial trabeculae were considered part of the RV cavity. After automatically tracking RV contours over the entire cardiac cycle using 3D technology, we obtained the RV end-diastolic and end-systolic volume (RV EDV and ESV, respectively), RVEF, and stroke volume (SV). Furthermore, we derived both the RV-free lateral wall (RVLS Free) and the interventricular septum (RVLS Septal) 3D STE longitudinal Strain (LS).

### 2.3. The ^1^H-NMR Spectroscopy

An expert operator centrifuged at 4000 rpm for 15 min at 4 °C the heparinized blood samples immediately after the collection, then stored the supernatant at −80 °C. Eight hundred μL of plasma was centrifuged at 4500 rpm for 10 min at 4 °C; four hundred μL of plasma was thawed and then subjected to extraction in chloroform/methanol solution of 1.2 mL of chloroform/methanol 1:1 in 175 μL of H_2_O, which was centrifuged at 4500 rpm at 4 °C for 30 min; the lipophilic and hydrophilic components, separated after centrifugation, were dried using an Eppendorf concentrator (Hamburg, Germany) and stored at −80 °C until the time of NMR analysis.

For the NMR experiments, we used a Varian UNITY INOVA 500 spectrometer operating at 499.839 MHz for proton and equipped with a 5 mm double-resonance probe (Agilent Technologies, Santa Clara, CA, USA). The ^1^H-NMR spectra were acquired using the following parameters: 300 K with a spectral width of 6000 Hz, a 90° pulse, an acquisition time of 2 s, a relaxation delay of 2 s, and 256 scans. To suppress the residual water signal, we applied a CPMG pre-saturation technique.

Obtained ^1^H-NMR spectra were pre-processed using ACDlab Processor Academic Edition (Advanced Chemistry Development, version 12.01, 2010) with 0.1 Hz line broadening that was zero-filled to 64 K and Fourier transformed. Subsequently, each spectrum was phased and baseline corrected, and the chemical shifts were referred to trimethylsilylpropanoic acid (TSP) peak level resonance at 0.00 ppm. The operator reduced ^1^H-NMR spectra into consecutive integrated spectral regions—named “bins”—of equal width (0.04 ppm) corresponding to the region 0.50–8.66 ppm. To remove the variation effect in the pre-saturation of the residual water resonance, we excluded the spectral region between 4.74 and 4.94 ppm from the analysis. Furthermore, to minimize the effects of the variable concentration among different samples, we normalized the integrated area within each bin to a constant sum of 100 for each spectrum. Finally, the obtained dataset was imported into SIMCA-P (version 14.0. Umetrics, Malmo, Sweden) program and Pareto scaled prior to analysis.

### 2.4. GC/MS

We centrifuged all study participants’ specimens at 2000 rpm for 10 min, then transferred supernatant in Eppendorf tubes and stored it at −80 °C until analysis. Plasma samples were thawed at room temperature. To form a pooled sample for quality control and an average composition sample to analyze, we collected 100 μL of each sample. An amount of 400 μL of plasma was treated with 1200 μL of cold methanol in 2 mL Eppendorf tubes, which was vortex mixed and centrifuged for 10 min at 14,000 rpm (16.9 G). An amount of 400 μL of the upper phase was transferred in glass vials (1.5 mL) and evaporated to dryness overnight in an Eppendorf vacuum centrifuge (Eppendorf SE, Hamburg, Germany). An amount of 50 μL of a 0.24 M (20 mg/mL) solution of methoxylamine hydrochloride in pyridine was added to each vial; samples were vortex mixed and left to react for 17 h at room temperature in the dark. Then, 50 μL of MSTFA (N-Methyl-N-Trimethylsilyltrifluoroacetamide) was added and left to react for 1 h at room temperature. As internal standard, the derivatized samples were diluted with hexane (100 μL) with tetracosane (0.01 mg/mL), just before GC/MS analysis [9]. Samples were analyzed using an Agilent 5975C interfaced to the GC 7820 (new 5977B/7890B) equipped with a DB-5 ms column (J&-W), with an injector temperature at 230 °C, detector temperature at 280 °C, and a helium carrier gas flow rate of 1 mL/min. The GC oven temperature program was 90 °C initial temperature with 1 min hold time and ramping at 10 °C/min to a final temperature of 270 °C with 7 min hold time. An amount of 1 μL of the derivatized sample was injected in split (1:4) mode. After a solvent delay of 3 min, mass spectra were acquired in full scan mode using 2.28 scans/s with a mass range of 50–700 Amu [9].

Each acquired chromatogram was analyzed using the free software AMDIS (Automated Mass Spectral Deconvolution and Identification System; http://chemdata.nist.gov/mass-spc/amdis, accessed on 25 November 2020) that identified each peak by comparison of the relative mass spectra and the retention times with those stored in an in-house library comprising 255 metabolites. Other metabolites were identified using NIST08 (National Institute of Standards and Technology’s mass spectral database) and the Golm Metabolome Database (GMD, (http://gmd.mpimp-golm.mpg.de/), accessed on 25 November 2020). Through this approach, 113 compounds were accurately identified, while 28 other metabolites were tentatively assigned by relying on GMD and NIST libraries. AMDIS analysis produced an Excel datasheet that was successively subjected to chemometric analysis [9].

### 2.5. Multi-Variate Statistical Analysis

We applied partial least square analysis (PLS), a supervised regression analysis method, and partial least square discriminant analysis (PLS-DA), which uses a Y-matrix containing information about the a priori class to which the sample belongs and which is used to evaluate the statistical significance of the classification [10].

### 2.6. Univariate Statistical Analysis

We compared continuous variables (anthropometric, clinical-laboratory, echo-cardiographic data) using the two-tailed *t*-test for non-paired samples and the exact Fisher test for the categorical ones, wherein a corrected value of two-tailed *p* < 0.05 was considered statistically significant. The analyses were performed using IBM SPSS v. 25.

## 3. Results

The anthropometric and clinical data of the population are shown in Table 1.

The average age of the enrolled population was 45.6 ± 12.2 years, with height and weight within the general population range (164 ± 7 cm and 62.9 ± 11.4 Kg, respectively) and a normal BMI (23.4 ± 4.3 Kg/m^2^). All SLE patients met the 2012 International Collaborating Clinics (LICUS) criteria [11].

Detailed clinical history was collected, including disease duration, current use, and cumulative glucocorticoid dosing; patients did not use other anti-inflammatory drugs except COX-2 inhibitors as needed. Cumulative organ damage was calculated with the Lupus International Collaborating Clinic/American College of Rheumatology (SDI) systemic damage index [12,13].

### 3.1. Standard and Advanced Echocardiography

**Figure 1:**
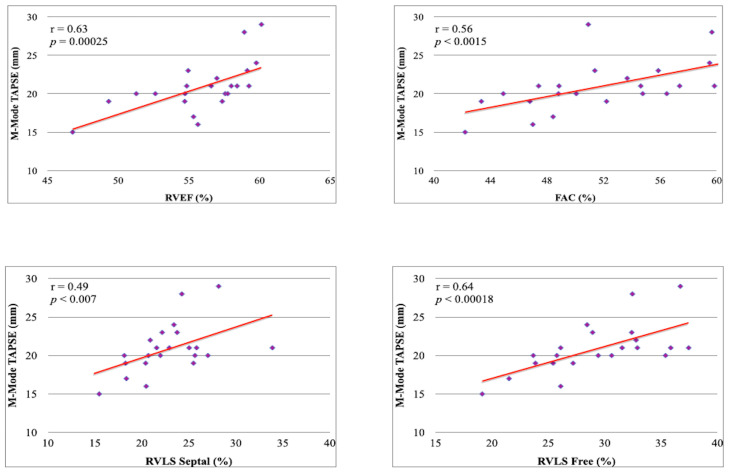
Correlation between M-Mode-Derived TAPSE and 3D RV systolic parameters.

**Figure 2:**
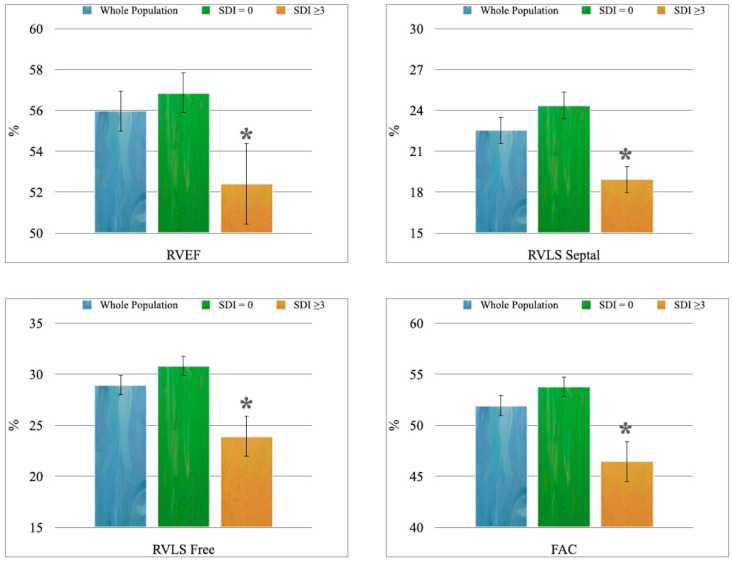
The 3D-echocardiographic parameters of RV systolic function: * *p* < 0.01 vs. “SDI = 0” group.

1. The patients, examined as a whole, showed normal echocardiographic parameters (Table 2), with normal LV size, wall thickness, and systolic function, a normal diastolic function or impaired relaxation, and regular left atrium dimensions. In addition, the standard parameters of the right sections were found to be normal in all the patients, except for the M-Mode TAPSE, which was reduced in the subjects with a higher SDI.

2. The 3D RV evaluation showed values aligned with the literature for SLE patients (Figure 1) [4]. We also obtaining a high significative direct correlation between the M-Mode-Derived TAPSE values and the parameters of RV systolic function evaluated by 3D ultrasound (Figure 1).

3. Subsequently, to evaluate the possible presence of RV dysfunction due to the underlying disease, we split the population according to the SDI values in patients with the absence of cumulative systemic damage (score = 0) and patients with more significant impairment (score ≥ 3).

4. Patients with higher cumulative damage showed statistically significantly lower RVEF, RVLS septal, RVLS Free, and FAC values than individuals with minimal impairment (Figure 2).

### 3.2. Metabolomics

^1^H-NMR: The metabolomic analysis highlighted a statistically significant correlation between the metabolism of the enrolled subjects and the RVLS septal, while the correlation with the RVLS Free did not reach significance (*p* = 0.09).

Subsequently, we verified whether the division into two groups carried out based on SDI values allowed us to identify different metabolic profiles related to the degree of RV dysfunction identified with 3D echocardiography; the built OPLS-DA model showed good values of R^2^ (R^2^_X_ = 0.529; R^2^_Y_ = 0.926) and Q^2^ (0.635), which were confirmed by ANOVA cross-validation (*p* = 0.04).

**Figure 3:**
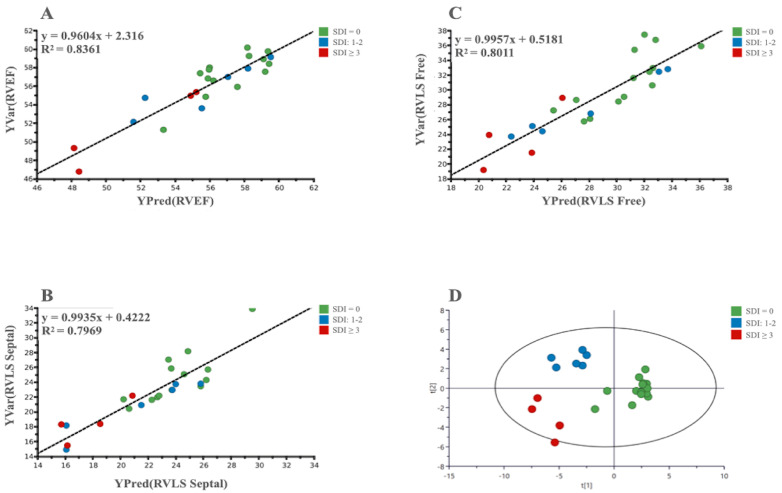
(**A**–**C**): PLS scatter plots show the correlation between metabolomic and 3D parameters of selected RV systolic function; (**D**): A 3-class OPLS-DA scatter plot showing the clustering of the metabolic data of patients based on the degree of organ damage evaluated by SDI value.

GC/MS: Analyses carried out using GC/MS allowed us to find significant correlations between the metabolism of LES patients and several 3D RV systolic parameters: RVEF, RVLS Free, and RVLS Septal (Figure 3).

In addition, in the GC/MS-based analysis, an OPLS-DA was able to identify SLE patients with the absence or severe degrees of both RV dysfunction and organ damage (Figure 3). Moreover, a three-class PLS-DA also highlighted a significant clustering in three groups based on the SDI score: Low (0), Intermediate (1–2) and High (≥3). All the analyses showed, beyond the significant *p*-value, good values for the R^2^ and Q^2^ (Table 3).

For every PLS-DA, we evaluated the variables important in prediction (VIP); the metabolites that were significantly involved in the observed correlations and clustering both at the multi-variate and univariate analyses were: 2,4-dihydroxybutyric acid, 3,4-dihydroxybutyric acid, citric acid, glucose, glutamine, glycine, linoleic acid, oleic acid, phosphate, urea, and valine (Table 4).

Overall, these results allow us to hypothesize that SLE causes progressive RV dysfunction, which was correlated to the severity of the cumulative damage and associated with a specific metabolic fingerprint.

## 4. Discussion

RV failure represents the leading cause of morbidity and mortality in SLE patients and substantially impacts the quality of life and prognosis in these subjects.

Buonauro et al. already demonstrated a reduction in the 3D FE, RVLS septal and RVLS free values in SLE patients compared with healthy controls [4]. In our study, we observed an alteration in the values of the 3D RVFE, both for the RVLS septal and RVLS free, as well asthe FAC. In addition, all these RV systolic indices derived from 3D echocardiography were directly correlated to the M-Mode-Derived TAPSE.

It is known how the TAPSE correlates with the RVFE measured with the scintigraphic method and with right cardiac catheterization [14,15], and its prognostic value has been ascertained [16,17,18,19]; the observed strong correlation with the parameters derived from 3D ultrasound confirmed the reliability of these data and strengthens their clinical significance.

In line with these data, the evidence of a more significant RV function impairment in patients with higher SDI values was found, which, therefore, led to a higher burden of organ damage. In addition, the reduction in TAPSE measured with the M-Mode in the SDI ≥ 3 group was associated with the reduction in systolic function parameters, both of those based on the study of size (FAC and RVFE) and those derived from myocardial deformation (RVLS septal and RVLS free) [4].

Tektonidou et al. found diastolic dysfunction of the RV and antibodies syndrome in SLE patients, regardless of the presence of valvular disease and RV systolic dysfunction [20]. In another study carried out in patients with SLE and PH, RV systolic function parameters were smaller than in control subjects and SLE patients who were free of PH [21].

Leal et al., using 2D echocardiography and LS evaluation, identified a subclinical systolic dysfunction of the RV in children with childhood-onset SLE [22].

Despite these exciting results, 3D ultrasound is superior to 2D; in fact, the 3D volumetric reconstruction of cardiac structures is not based on geometric assumptions, but on the direct measurement of volumes and, therefore, of the EF. Quantifying RV geometry and function using 3D ultrasound was reproducible and accurate, even when compared with cardiac MRI [23,24].

Vitarelli et al. demonstrated the prognostic value of 3D RVEF in patients with acute pulmonary embolism, which is a clinical condition in which increased pulmonary pressures are often present [25].

Although few studies have evaluated the association between 3D LS and clinical outcomes, its feasibility has also been confirmed in clinical practice [26].

Also, in our study, the 3D evaluation allowed for unmasking an early longitudinal dysfunction of the RV that was correlated with the severity of the patients’ global organ damage.

Pieretti et al. had already shown that, in SLE patients without clinical or echocardiographic evidence of valvular or coronary artery disease, the SDI could predict an increase in LV mass [27]. The SDI was associated with RV damage in both Buonauro et al.’s and our studies [4]. Compared to the work of the Neapolitan group, in which RV involvement was only identifiable using the 3D STE technology, but not through the standard (TAPSE and tricuspid E/A ratio) or 3D volumetric (EF) parameters, our results show a reduction in the RVEF, FAC and TAPSE; this difference is likely due to the different SDI cut-off values used to divide patients: ≥1 in the case of Bonauro et al., ≥3 in ours.

The finding of early and subclinical impairment of the RV systolic function is not simple instrumental data; it represents a critical issue, since RV dysfunction is associated with poor prognosis and higher mortality risk in comparison with subjects with a preserved systolic capacity, as was already highlighted in patients affected by PH [28].

Moreover, both left and right ventricular dimensions and function modifications correlate with prognosis [29,30] and response to therapies [31,32].

On these bases, the evaluation of the RV is of outstanding importance and could become a routine practice for cardiologists in the setting of both primary and secondary cardiovascular diseases. In the setting of SLE, the degree of longitudinal dysfunction of the RV is, on the whole, associated with the extent of organ damage measured with the SDI.

Inflammation is a biological defense system, which is essential in response to pathogenic noxae, such as infections or tissue damage, and it is supported by the activity of the immune system in order to ensure survival. Inflammatory responses may be considered as part of a classic homeostatic system, which maintains the normal function of organs and systems [33].

In the heart, the activation of “sterile” inflammatory processes is mainly similar to that observed during infections: this involves the release of vasoactive peptides; the expression in cardiac cells (cardiomyocytes, fibroblasts, endothelial cells) of adhesion molecules (such as vascular cell adhesion molecule (VCAM)-1 and intercellular adhesion molecule (ICAM)-1), which promote the myocardial recruitment of inflammatory cells (neutrophils, macrophages, lymphocytes); the release of inflammatory cytokines and chemokines; and the activation of adaptive immune responses mediated by T cells [34,35,36,37].

The activation of inflammatory processes in the RV is associated with and contributes to its remodeling and progression to dysfunction [33,38,39].

In RV failure, a high expression of cytokines and chemokines modulates numerous intracellular pathways in cardiac cells, thus leading to hypertrophy and the death of cardiomyocytes, mitochondrial dysfunction, endoplasmic reticulum stress, and cardiac fibrosis, which is characterized by fibroblastic proliferation and differentiation, as well as collagen deposition [35,37].

Furthermore, these inflammatory mediators can also alter the myocardium’s metabolic processes and the cardiomyocytes’ contractile properties.

This data seems to be confirmed by the results of the metabolomic analysis, which showed a direct correlation between RV systolic parameters, such as EF and LS, and the metabolism of SLE patients.

Our group had already demonstrated a correlation between the LV longitudinal systolic function, assessed by the longitudinal strain rate, and the metabolic profile of healthy subjects and patients suffering from heart failure of increasing degrees [40].

Few works in the literature evaluate metabolism in pulmonary hypertension and RV failure; our study is the first in humans to investigate the correlation between metabolism and RV systolic function in SLE patients who are PH-free, with traditional parameters of systolic function that are still within normal limits, but with echocardiographic evidence of subclinical RV dysfunction.

The correlation between the RVEF and RVLS septal and the RVLS free and metabolism suggests that, even in the RV, the biogenesis of chemical energy is fundamental for maintaining contractile function. It is well known that myocardium metabolism is based, in physiological conditions, on the preferential use of free fatty acids, while, in the course of the disease, it switches to the consumption of glucose [41].

In line with this hypothesis, various studies have shown impairment in the energy metabolism of the RV over the course of hypertrophy/failure.

RV maladaptive alterations involve aerobic glycolysis (resulting from a transcriptional upregulation of pyruvate dehydrogenase kinase) and glutaminolysis (which reflects the activation of cMyc induced by ischemia) [42].

Accordingly, our findings show the importance of glucose, fructose, citric acid, glutamine, ketone bodies, oleic, and linoleic acids in determining the clustering of impairment degree and the correlations with the echocardiographic parameters of RV systolic function.

Overall, these findings seem to point out a disarrangement of the energetic metabolism of the RV, and it has already been demonstrated that, in experimental RV hypertrophy, the glycolytic metabolic shift is associated with RV dysfunction [43]. This metabolic switch that shunts glucose metabolites from the mitochondria to glycolysis to maintain ATP production is common to what happens in left ventricular heart failure [6], and it seems to prevent toxic ROS production [44].

More recently, Fowler’s group demonstrated that an altered functioning of the creatine kinase system is present in the decompensated RV and that an improvement in energy metabolism could be one of the causes of the better survival of rats with PH that were treated with beta-blockers [45]. In line with this observation, our analysis highlighted the importance of glycine, an amino acid involved in creatine synthesis, that demonstrated a protective effect against reoxygenation injury in the mitochondria of cardiomyocytes subjected to ischemia or Ca^2+^ stress under normoxia [46]; furthermore, this metabolite could have a prominent role in preserving energy production in the mitochondria of myocytes during acute cellular stress [47].

Another metabolite that suggests an energetic metabolism alteration is the urea, whose cycle upregulation can increase the contribution of arginine to the Krebs cycle through the generation of fumarate, which acts as a Krebs cycle intermediate [48]; in addition, the urea cycle was involved in the development of PH in rats [49].

In this context, cytokines play a crucial role [50]: among these, interleukin (IL)-1 [51], tumor necrosis factor (TNF)-α [52], and IL-6 [53] are involved in SLE pathophysiology, but they are also involved in the development and progression of RV dysfunction [33].

## 5. Conclusions

In conclusion, our study confirms the efficacy of 3D echocardiography in identifying RV function subclinical changes in SLE patients, especially in the absence of clinical evidence of cardiovascular disease. Furthermore, the alteration of RV systolic function, which underlies the existence of subclinical damage to the RV myocardium, correlates with SLE patients’ metabolisms, and the latter results are different based on the SDI values.

The ability of the OPLS-DA to discriminate between the groups based on the SDI score, together with the correlation between metabolism and RV systolic function parameters, seem to suggest that the subclinical dysfunction identified in SLE patients results from myocardial damage that is induced through various mechanisms, including oxidative stress and by inflammatory cytokines; this damage would subsequently be superimposed on alterations in energy metabolism that would likely be similar to those observed in left ventricular decompensation, which would perpetuate and aggravate RV dysfunction.

## Figures and Tables

**Table 1:** Anthropometric and clinical data of the study population.

Parameter	Whole Population	SDI = 0	SDI ≥ 3	*p*-Value
Age (years)	45.6 ± 12.2	42.9 ± 12.6	55.8 ± 10.6	NS
Male sex (%)	10.3	12.5	20	NS
Height (cm)	164 ± 7	165 ± 9	161 ± 7	NS
Weight (Kg)	62.9 ± 11.4	64.7 ± 12.5	62.6 ± 12.2	NS
BMI (Kg/m^2^)	23.4 ± 4.3	23.7 ± 4.5	24.1 ± 4.7	NS
CKD > stage 3	1	0	1	NS
Proteinuria	3	3	0	NS
QRISK3 score	11.7 ± 16.5	6.5 ± 4.0	24.8 ± 7.5	0.03
Prednisone (mg)	9.2 ± 8.8	8.1 ± 11.4	10.1 ± 7.5	NS
SLEDAI	6.0 ± 4.6	5.4 ± 5.1	5.8 ± 5.3	NS

BMI: Body mass index. CKD: Chronic kidney disease. SLEDAI: Systemic lupus erythematosus disease activity index. NS = Non-Significant.

**Table 2:** Echocardiographic parameters of the study population.

Parameter	Whole Population	SDI = 0	SDI ≥ 3	*p*-Value
EDD (mm)	44.9 ± 4.1	45.0 ± 0.7	43.4 ± 3.6	NS
IVS (mm)	8.4 ± 1.1	8.4 ± 1.2	9.1 ± 0.8	NS
PW (mm)	8.8 ± 1.3	8.5 ± 1.4	9.4 ± 1.1	NS
2D-LVEF (%)	64.6 ± 4.4	64.5 ± 4.5	65.4 ± 5.6	NS
3D-EDVI (mL/m^2^)	57.6 ± 4.7	57.3 ± 5.1	53.6 ± 4.9	NS
3D-ESVI (mL/m^2^)	20.7 ± 3.9	20.9 ± 4.7	19.2 ± 4.2	NS
3D-LVEF (%)	63.9 ± 5.1	63.5 ± 5.5	64.2 ± 4.8	NS
LAVI (mL/m^2^)	26.9 ± 5.8	24.6 ± 5.4	30.7 ± 6.4	NS
E/E′	7.9 ± 1.5	7.8 ± 1.3	8.9 ± 1.2	NS
RV EDD (mm)	30.8 ± 5.2	30.4 ± 2.8	35.5 ± 10.4	NS
RV EDV (mL)	54.7 ± 14.1	54.3 ± 8.9	56.2 ± 26.2	NS
RV ESV (mL)	24.6 ± 6.3	24.4 ± 3.9	27.5 ± 15.4	NS
RV S′ (cm/s)	12.0 ± 2.3	12.7 ± 1.8	11.3 ± 2.6	NS
TAPSE (mm)	20.9 ± 3.3	21.7 ± 3.1	18.0 ± 3.1	<0.05
Right Atrium area (cm^2^)	13.0 ± 2.8	12.6 ± 2.2	14.5 ± 4.6	NS
PAP (mmHg)	25.2 ± 11.4	23.6 ± 5.4	36.7 ± 23.1	NS
SV/ESV	1.28 ± 0.16	1.33 ± 0.14	1.11 ± 0.17	<0.01
TAPSE/PAP (mm/mmHg)	0.83 ± 0.13	0.92 ± 0.08	0.49 ± 0.26	<0.01

EDD: End-Diastolic diameter. IVS: Interventricular septum. PW: Posterior wall. LVEF: Left ventricular ejection fraction. EDVI: End-Diastolic volume index. ESVI: End-Systolic volume index. LAVI: Left atrium volume index. RV TDD: Right ventricular end-diastolic diameter; RV EDV: Right ventricular end-diastolic volume. RV EDV: Right ventricular end-systolic volume. TAPSE: Tricuspid annular plane systolic excursion. PAP: Pulmonary artery pressure. NS = Non-Significant.

**Table 3:** Parameters of the OPLS-DA models derived from LES patients’ plasma samples and relative *p*-values evaluated by ANOVA.

Multi-Variate Statistical Models	RVEF	RVLS Septal	RVLS Free
R^2^X	0.863	0.797	0.801
Q^2^	0.464	0.489	0.392
*p*-value (CV-ANOVA)	0.01	0.005	0.022

**Table 4:** List of the most significant metabolites obtained by multi-variate and univariate statistical analysis.

Metabolite	VIP Value	*p*-Value
2,4-dihydroxybutyric acid	1.23512	≤0.002
3,4-dihydroxybutyric acid	1.53081	≤0.002
Citric acid	1.15949	≤0.02
Glutamine	1.0001	≤0.05
Glucose	1.19069	≤0.001
Glycine	1.46283	≤0.0001
Linoleic acid	1.23842	≤0.0001
Oleic acid	1.00864	≤0.0001
Phosphate	1.13964	≤0.0001
Urea	2.10386	≤0.02
Valine	1.12079	≤0.02

## Data Availability

The datasets used and analyzed during the current study are available from the corresponding author upon reasonable request due to ongoing analyses.
